# Lack of evidence on mental health and well-being impacts of individual-level interventions for vulnerable adolescents: systematic mapping review

**DOI:** 10.1016/j.puhe.2018.04.003

**Published:** 2018-08

**Authors:** G. Vojt, K. Skivington, H. Sweeting, M. Campbell, C. Fenton, H. Thomson

**Affiliations:** MRC/CSO Social and Public Health Sciences Unit, Institute of Health and Wellbeing, University of Glasgow, 200 Renfield Street, Glasgow G2 3QB, United Kingdom

**Keywords:** Vulnerable adolescents, At-risk adolescents, Mental health, Health inequalities, Systematic review, Mapping review

## Abstract

**Objectives:**

To review empirical evaluations of individual-level interventions intended to improve mental health or well-being for vulnerable adolescents.

**Study design:**

This is a systematic mapping review.

**Methods:**

Thirteen databases covering academic and gray literature were searched for published reviews and randomised controlled trials, and gray literature (2005–2016) and the results quality-assessed to prioritise best available evidence. We aimed to identify well-conducted systematic reviews and trials that evaluated individual-level interventions, for mental health/well-being outcomes, where the population was adolescents aged 10–24 years in any of 12 vulnerable groups at high risk of poor health outcomes (e.g. homeless, offenders, ‘looked after’, carers).

**Results:**

Thirty systematic reviews and 16 additional trials were identified. There was insufficient evidence to identify promising individual-level interventions that improve the mental health/well-being of any of the vulnerable groups.

**Conclusions:**

Despite Western policy to promote health and well-being among vulnerable young people, the dearth of evidence suggests a lack of interest in evaluating interventions targeting these groups in respect of their mental health/well-being outcomes.

## Highlights

• Adolescence is a critical life course period for interventions to reduce/prevent current and future distress and dysfunction.
• Alongside population health approaches, targeted approaches to high-risk groups are valuable to address health inequalities.
• We found insufficient evidence of individual-level interventions to improve mental health/wellbeing of vulnerable groups.
• Despite policy, our review suggests a lack of evaluation of mental health/wellbeing outcomes of such targeted interventions.

This short communication reports on findings^1^ in response to a call from the Royal Society of Edinburgh Scotland Foundation to conduct a systematic review of empirical evaluations of individual-level interventions intended to improve mental health, happiness or well-being or reduce health inequalities for young people undergoing the transition to adulthood. The population was vulnerable adolescents, and the UK political context is national guidelines and policies vowing to safeguard this group. For governments to be able to do this, evidence on effective intervention is required.

Adolescence is a critical period in the life course, encompassing many changes related to biological and psychosocial development likely to impact on transitions into adulthood. It is also when most mental health disorders begin, associated in turn with negative health, behavioural and educational outcomes, and impacting relationships with family and friends and the ability to develop independence.^2^ The transition into adulthood is likely to be more challenging for the most vulnerable and disadvantaged young people, who are at high risk of poor health, educational, behavioural and relationship outcomes and likely to require additional support to make successful and healthy transitions. Adolescence is, therefore, a key life stage for mental health–related interventions aiming to reduce/prevent current and future distress and dysfunction.^2^ Interventions aimed at high-risk groups represent a valuable component of strategies to address health inequalities, complimentary to population health approaches.^3^ Because most population-based interventions for adolescents are delivered in a school setting, they are likely to miss many vulnerable groups, particularly those who do not attend school on a regular basis, and a blanket approach is unlikely to tap into the complex needs of vulnerable young people, for example, the study by Schofield and Simmonds.^4^

We conducted a systematic mapping review, for which the protocol is available.^5^ This approach aims to map out, categorise, and identify gaps in research literature.^6^ We systematically reviewed English language studies and reviews conducted in Organisation for Economic Co-operation and Development (OECD) countries, and published since 2005, to identify evidence in three different publication categories: reviews published in journals, randomised controlled trials (RCTs) published in journals and evaluations including a comparison group in the gray literature. The beginning of 2005 was selected for pragmatic reasons, ensuring a manageable number of ‘hits’ representing recent relevant interventions (and assuming important earlier ones would be captured in reviews); we conducted the searches in 2016.

In line with the call to focus on interventions to reduce inequalities for young people undergoing the transition to adulthood, our systematic review specifically focused on ‘vulnerable’ adolescents. Our remit did not extend to studies conducted on clinical populations or examining the impact of interventions on disease end points. Although there is ‘no universally accepted definition of a ‘vulnerable group’’, the concept generally encompasses those who are ‘marginalised, socially excluded, have limited in opportunities and income and suffer abuse […] hardship, prejudice and discrimination’.^7^^(p3)^ It is also important to note that vulnerable group membership may be transient and/or that individuals may have overlapping vulnerabilities. The included groups (the population) were selected in consultation with an Expert Advisory Group and comprised:

• ‘Looked after’/care leavers
• Homeless
• Young offenders
• Sexually abused
• Teenage parents
• Ethnic minorities
• Asylum seekers/refugees
• Victims of domestic/intimate partner violence
• Living in socio-economically deprived areas
• Unemployed
• Out of/excluded from school
• Young carers

Within these groups, we included adolescents aged 10–24 years, a broad age range, encompassing roughly the start of secondary school to young adulthood to ensure we captured interventions likely to impact on the transition to adulthood. We excluded clinical populations under medical treatment or supervision.

Evaluations of individual-level interventions that aimed to improve mental health, well-being or happiness, or included one of these concepts (measures of general mental health, well-being, life satisfaction, happiness, resilience, impulsivity, self-esteem, sense of coherence) as a primary or secondary outcome were included. Interventions that were pharmaceutical or received in clinical or school settings were excluded. To be included, studies had to report on a comparison, either a control group or before and after measures of the outcome, enabling us to make some evaluation of the intervention. In systematic reviews, we assessed the number of studies relevant to our inclusion criteria to ensure that the conclusions were applicable to our review question.

A broad search strategy was used that included a combination of appropriate keywords, medical subject headings and free-text terms. We conducted our searches across 12 databases: MEDLINE; Embase; British Education Index; PsycARTICLES; SocINDEX; ERIC; Child Development & Adolescent Studies; Social Care Online; PsycINFO; Cochrane Library; Campbell Library; and Planex (full search strategy including inclusion and exclusion criteria available^2^). Two authors independently screened 10% of the search results at the title/abstract stage to ensure reviewer consistency. All reviews/studies that proceeded to full-text stage were independently screened by two authors. Disagreements were resolved by consensus via a third reviewer. To prioritise best available evidence, our conclusions were based on well-conducted systematic reviews and RCTs. Included systematic reviews were appraised for quality using an amended version of the AMSTAR tool.^8^ A structured data extraction template was completed for each review/study by one author and checked by a second author for consensus. Narrative synthesis of findings was conducted.

Our search identified 7231 systematic reviews and 4449 RCTs. After screening, 30 reviews (20 rated high quality) and 16 RCTs were included. No relevant evaluations with a control group were identified in the gray literature. Table 1 summarises identified evidence for each population group. Importantly, there was insufficient evidence to identify interventions which clearly benefit the mental health/well-being of any of the included vulnerable groups. Overall, evidence was either conflicting, absent or too limited to enable clear effectiveness statements. However, a small body of evidence was identified which reported some evidence on mental health impacts for some groups.

**Table 1:** Summary of identified evidence across all vulnerable populations.

Vulnerable group	Systematic review—high quality (n)		Systematic review—low quality (n)	Randomised controlled trials (n)	Gray literature evaluations (n)
	Reviews	Relevant studies in each review			
**Some evidence identified**					
Looked after	7	11	3	3	0
Homeless	3	20	1	1	0
Young offenders	3	14	1	0	0
Teenage parent	2	0^a^	0	7	0
Experience of sexual abuse	2	11	2	1	0
**Very little evidence identified**					
Asylum seeker/refugee	1	1	0	1	0
Ethnic minority	1	0^a^	0	1	0
Exposure to domestic violence or intimate partner violence	0	–	1	1	0
Living in socio-economically deprived areas	1	0^a^	2	1	0
**No evidence identified**					
Unemployed	0	–	0	0	0
Out of school/excluded	0	–	0	0	0
Young carers	0	–	0	0	0
**Total**	**20**		**10**	**16**	**0**

a Some systematic reviews failed to identify any studies with outcomes pertaining to mental health/well-being.

Available evidence suggests cognitive behavioural therapy can benefit the mental health of homeless adolescents, young offenders and adolescents who have been sexually abused. Evidence on the mental health impact of psychological interventions for ‘looked after’ adolescents or teenage parents is limited. There is some evidence that practical support services can benefit the mental health of homeless adolescents.

Very little evidence (a single study or high-quality systematic review) was identified evaluating the mental health/well-being impacts of interventions targeting asylum seekers/refugees; ethnic minorities; adolescents exposed to domestic/interpartner violence; or those living in socio-economically deprived neighbourhoods. We identified no evidence for three groups: unemployed; out of school/excluded; and young carers.

Thus, our systematic mapping review identified a remarkable lack of evidence in respect of individual-level interventions to improve the mental health/well-being of (non-clinical) vulnerable adolescents. It was the near total absence of research, rather than the specifics of particular interventions for the different groups, which we think is a particularly striking finding from this work.

One potential reason for our lack of findings is that we somehow failed to identify the full range of interventions being implemented in practice. We focused on reviews and RCTs rather than broader sources of research evidence including non-randomised and/or qualitative studies. However, we conducted comprehensive searches of relevant literature and consulted our Expert Advisory Group to ensure appropriate coverage of the field; no important gaps were highlighted. Another possibility is that mental health/well-being interventions with vulnerable adolescents have focused on diagnosed psychiatric disorders (which our review specifically excluded), rather than a broader or more salutogenic perspective. In addition, as became clear to us, many of the interventions targeted at vulnerable adolescents who address determinants of health (e.g. employment; homelessness; reoffending) and may have been evaluated for effectiveness in achieving these outcomes, but largely overlooking potential mental health/well-being outcomes.

The vulnerable adolescents included in our systematic mapping review are both at high risk of poor mental health and at a critical period in their lives. Governments, including the UK's, have committed this group as a priority.^9^, ^10^ However, to improve health/well-being outcomes for society's most vulnerable young people, a key step in reducing health inequalities, is to include targeted individual-level interventions to compliment universal mainstream interventions.^3^ In a context of policy and national guidelines focussing on safeguarding vulnerable adolescents, there is still a need for more research on what individual-level interventions work to promote their mental health/well-being. The focus of both policy and research appears to have been on interventions, addressing socio-economic determinants of health. There is a need for researchers, policy makers and practitioners to think more holistically and routinely consider mental health/well-being as key outcomes when implementing interventions aiming to improve the social circumstances of vulnerable adolescents. In addition to other factors not considered here (e.g. greater understanding of those who are unidentified and/or slip through the net and so do not receive the interventions they need), such evidence would inform future investment to improve mental health and well-being among vulnerable adolescents and facilitate a successful transition into adulthood. In doing so, it would impact on the future of lives of the most disadvantaged young people and subsequent generations.

## Author statements

### Ethical approval

Ethical approval was not required for this review.

### Funding

This work was commissioned and funded by an external grant from the Royal Society of Edinburgh Scotland Foundation. KS, HT, HS, and MC are funded by Medical Research Council Grants (MC_PC_13027 & MC_UU_12017/15 & MC_UU_12017/12) and Chief Scientist Office of the Scottish Government Health Directorates Grants (SPHSU12 & SPHSU15).

### Competing interests

The authors declare no competing interests.
